# Contribution of Fruit, Vegetables, Whole Cereals, and Legumes To Total Fibre Intake in Adult Croatian Dalmatian Population

**DOI:** 10.2478/aiht-2020-71-3305

**Published:** 2020-06-29

**Authors:** Marijana Matek Sarić, Marija Ljubičić, Ivana Lapčić, Raquel Pinho Ferreira Guiné

**Affiliations:** 1 University of Zadar, Department of Health Studies, ZadarCroatia; 2 General Hospital Zadar, Department of Paediatrics, ZadarCroatia; 3 Medical School Ante Kuzmanića, ZadarCroatia; 4 Polytechnic Institute of Viseu, Campus Politécnico, Viseu, Portugal

**Keywords:** conversion factors, dietary fibre, fibre-rich food, recommended dietary requirements, TFI, faktor pretvorbe, hrana bogata vlaknima, prehrambena vlakna, preporučene prehrambene potrebe, TFI

## Abstract

There is compelling evidence that fruit, vegetables, whole cereals, and legumes make about 80 % of the total food fibre intake and have a potential to help in the prevention of a number of diseases. The aim of our study was to estimate total fibre intake from consumption of this fibre-rich food, partly reported in our earlier study in Croatian adult population. Current data analysis involved a non-probabilistic sample of 1,034 adult participants from Dalmatia, Croatia who responded to a validated food frequency questionnaire between October 2014 and March 2015. We also analysed the sales data obtained from three shopping centres in the Zadar area (Croatian coast) to establish a list of most frequently bought fruit, vegetables, whole cereals, and legumes and to calculate dietary fibre (DF) intake for each of the top-selling items and conversion factors for each food group. We then used these conversion factors to calculate individual total fibre intake (TFI) in our population. It was 11.4 g per person per day, which is less than half the recommended dietary requirements. On average, respondents reported to consume one piece of fruit and one meal of vegetables a day, which is less than half the daily recommendation for either. 25.8 % of respondents reported no consumption of whole cereals at all, and only 0.2 % of the population consumed the recommended 3–5 servings of whole grains or legumes a day. We also observed significantly higher consumption of fruit and whole grains/legumes in women than men. Our findings alert to poor dietary fibre intake in Croatian adult population, which is similar to other western countries and points to issues deeply rooted in these economies. However, our findings may be either an over- or under-estimation and need to be verified through longitudinal research on a wider sample using more precise tools.

## Abstract (HR)

Postoje uvjerljivi dokazi da su voće, povrće, cjelovite žitarice i mahunarke glavni izvori prehrambenih vlakana iz hrane, tj. čine oko 80 % ukupnog unosa prehrambenih vlakana i mogu potencijalno pomoći u prevenciji mnogih bolesti. Cilj naše studije bio je procijeniti ukupni unos vlakana konzumacijom hrane koja predstavlja glavne izvore prehrambenih vlakana. Dio podataka objavljen je u našoj ranijoj studiji za odraslu hrvatsku populaciju. Konkretna analiza podataka uključivala je uzorak od 1.034 odrasla ispitanika u Dalmaciji u Hrvatskoj koji su odgovorili na validirani upitnik o frekvenciji unosa hrane bogate vlaknima između listopada 2014. i ožujka 2015. Također, analizirali smo i uspostavili popis najčešće kupljenog voća, povrća, cjelovitih žitarica i mahunarki u trima trgovačkim centrima na području grada Zadra (hrvatska obala). Na osnovi dobivenih podataka za svaki smo pojedinačni proizvod izračunali čimbenik pretvorbe te ga procijenili za pojedinu skupinu hrane: voće, povrće, cjelovite žitarice i mahunarke. Dobivene čimbenike pretvorbe te podatke iz ankete upotrijebili smo za izračun ukupnog unosa vlakana (TFI) za našu ispitivanu populaciju, i on je iznosio 11,4 g po osobi dnevno, što je samo polovica preporučenih prehrambenih potreba. U prosjeku su ispitanici izvijestili da konzumiraju jedan komad voća i jedan obrok povrća dnevno, što je manje od polovine dnevne preporuke za oba izvora. Četvrtina ispitanika (25,8 %) izvijestila je da uopće ne konzumira cjelovite žitarice, a njih samo 0,2 % konzumiralo je preporučenih 3 – 5 obroka cjelovitih žitarica ili mahunarki dnevno. Također smo uočili značajno veću konzumaciju voća i cjelovitih žitarica/mahunarki kod žena u odnosu na muškarce. Naša otkrića upozoravaju na nedovoljan unos prehrambenih vlakana u odrasloj populaciji u Dalmaciji u Hrvatskoj, što je slično nalazima iz drugih zapadnih zemalja te upućuje na duboko ukorijenjene probleme u tim gospodarstvima. Međutim, naša otkrića mogu biti ili precijenjena ili podcijenjena, stoga ih je potrebno potvrditi novim longitudinalnim istraživanjima na širem uzorku, koristeći se preciznijim alatima.

Increasing evidence suggests that healthy eating with increased consumption of plant-based food prevents chronic diseases (1, 2), including non-communicable diseases (NCDs) (3), and has multiple benefits for human health. This effect is related their dietary fibre (DF) content, which includes non-digestible carbohydrates of three or more monomeric units, isolated or synthetic fibres with demonstrated physiological benefits (such as resistant starches), and minor components like lignin in plant cell walls (3, 4, 5, 6, 7, 8, 9, 10, 11, 12).

However, diet high in fruit, vegetables, legumes, and cereals is widely recommended not only as a DF source but also as a source of vitamins (C and A in particular), minerals (especially electrolytes), and phytochemicals (especially antioxidants), and it is very difficult to clearly distinguish the health benefits of these components separately (10, 13).

Even when it comes to total fibre intake (TFI) alone, little has been studied about how each of the these main sources of DF contribute to it and what dietary habits should be changed to achieve the desired TFI of 25–32 g for women and 30–35 g for men or alternatively 14 g of DF/1,000 kcal per day and capita (3, 14). This amount changes with gender, age, and recommending organisation, whether it is the United Nations World Health Organization (WHO), Food and Agriculture Organization (FAO), Associated Health Foundation (AHF), American Pediatric Association (APA), American Diabetes Association (ADA), European Food Safety Authority (EFSA), or National Academy of Sciences (Dietary Reference Intakes). A joint WHO/FAO/EFSA recommendation sets the minimum DF intake to 25 g/day (15, 16).

DF should be obtained from as varied sources as possible, because variety is the golden rule for healthy diet. Eating more fibre-rich food like vegetables, fruit, whole grains, and legumes as principal food fibre sources has been one of the WHO recommendations since 2003 (15). WHO recommends eating at least 400 g of fruit and vegetables a day, more specifically, 200 g of fruit (at least 2–3 servings) and 200 g (2–5 servings) of vegetables (17). The dietary guidelines of the US Whole Grains Council recommend at least 80 g (3–5 servings) of whole grains a day (17).

## Participants and methods

The first phase of this cross-sectional study included 1,034 Croatian adults between October 2014 and March 2015. Data that we present here were collected as part of a larger survey reported earlier (18) with the help of a validated, quick, and reproducible food frequency questionnaire (FFQ) (19) with 40 items to determine demographic characteristics and average daily and weekly intake of food rich in fibre (5, 20). The participants were asked to report their weekly consumption of each item (meal, serving, or portion). Responses (consumption frequency) were numeric values.

We recruited through newspaper advertisements, events at shopping and town centres, and word-of-mouth. The intention was to cover the whole range of ages and education levels among adult urban and rural population of both genders. The participants were informed about the purpose of the study and assured that participation was voluntary and anonymous. Upon signing consent, the participants filled out the survey in cubicles to ensure their privacy. The study was approved by the Human Research Ethics Committee of the General Hospital Zadar. All data were collected and analysed in accordance with the Declaration of Helsinki.

The second phase of the study, conducted in May 2018, involved the analysis of sales data for fruit, vegetables, whole grains, and legumes obtained from three shopping centres in the Zadar area (with a population of 70,000) to determine the top selling items for each food group. Based on DF content per 100 g of each specific food item established by the United States Department of Agriculture (USDA) National Nutrient Database (21) and portion size, we then calculated total fibre content for each item. Portions and servings were defined according to literature (22, 23, 24, 25, 35), while meal corresponded to one third or one fifth of all food (serving or portions) consumed a day (26, 27) Average servings were 30 g for nuts, 50 g for dried fruit, 150 g for fresh fruit, 200 g for vegetables, and 50–200 g for whole grains and legumes.

To calculate consumption in our population, DF intake in grams from each food source (fruit, vegetables, whole grain, and legumes) and total fibre intake we relied on the mathematical models described in detail by Tarcea et al. (28).

Conversion factors needed to calculate TFI were obtained as follows: we multiplied DF content of each item [available in reference (21)] on the list of top-selling produce (dried fruit included) with the weight of each serving/portion/piece to obtain total fibre content per serving/portion/piece. Then we averaged total fibre of all food items in a group (Tables 1–3). These averages served as conversion factors to calculate DF intake for each food group. Intake reported in the questionnaire was then multiplied with the conversion factor of 3.05 for fruit, 4.16 for vegetables, and 10.14 for cereals and legumes to obtain TFI.

**Table 1:** Total fibre in portions/pieces of fruit

Food group	Dietary fibre (g)/100g	Portions/pieces (g)	Total fibre (g)
**Fruit**			
Apricot	2.00	150	3.00
Banana (peeled)	2.60	150	3.90
Grapes	0.90	150	1.80
Kiwi	3.00	150	4.50
Lemon	2.80	150	4.20
Nectarine	1.70	150	2.55
Peach	1.50	150	2.25
Pear	3.10	150	4.65
Plum	1.40	150	2.10
Pineapple	1.40	150	2.10
Apple (with skin)	2.40	150	3.60
*Average for fruit*			3.15
**Dried fruit**			
Almond	12.5	30	3.75
Hazelnut	9.70	30	2.91
Pistachio	10.60	30	3.18
Walnut	6.70	30	2.01
Peanut	8.00	30	2.40
Dried Apricot	7.70	50	3.83
Dried cranberries	5.00	50	2.50
*Average for dried fruit*			2.94
Conversion factor for fruit			3.05

The amount of fibre (in g) in individual foods was taken from (34) and the usual portion /meal serving size (in g) was taken from (23, 25)

**Table 2:** Total fibre content in portion of vegetables

Food group	Dietary fibre (g)/100g	Meal serving (g)	Total fibre (g)
**Vegetables**			
Cabbage	2.50	200	5.00
Broccoli	3.30	200	6.60
Corn	2.40	200	4.80
Carrot	2.80	200	5.60
Cauliflower	2.00	200	4.00
Mangel/spinach	1.60	200	3.20
Lettuce	1.90	200	3.80
Pepper	1.70	200	3.40
Potatoes	2.50	200	5.00
Zucchini	1.00	200	2.00
Tomato	1.20	200	2.40
*Average for vegetables*			4.16
Conversion factor for vegetables			4.16

The amount of fibre (in g) in individual foods was taken from (34) and the usual portion /meal serving size (in g) was taken from (23, 25)

**Table 3:** Total fibre content in portions of whole cereals and legumes

Food group	Dietary fibre (g)/100g	Meal serving (g)	Total fibre (g)
**Cereals**			
Cornflakes	3.60	50	1.08
Brown rice	1.80	200	3.60
Wholemeal bread	6.80	50	3.40
Whole breakfast cereals	11.70	50	5.85
Oatmeal (uncooked)	10.60	50	5.30
Oatmeal (cooked)	1.70	200	3.40
Quinoa	2.80	200	5.60
Buckwheat	10.00	200	20.00
Corn flour/polenta	6.90	200	13.8
*Average for cereals*			6.89
**Beans**			
Chickpeas	12.20	150	18.30
Lens	10.80	150	16.20
Beans (cut green)	2.00	150	3.00
Beans dried	17.00	150	25.50
Peas (row)	2.60	150	3.90
*Average for beans*			13.38
Conversion factor for whole cereals and legumes			10.14

The amount of fibre (in g) in individual foods was taken from (34) and the usual portion /meal serving size (in g) was taken from (23, 25)

### Statistical analysis

The collected data were processed with SPSS Statistics for Windows, version 22.0 (IBM, Armonk, NY, USA). Normality of distribution was tested with the Kolmogorov-Smirnov test. For descriptive statistic we calculated medians and interquartile ranges for numeric variables as well as absolute numbers and percentages for categorical variables, because data distribution was not normal.

For the analysis of differences between men and women in total weekly consumption of fruit (in pieces or servings), vegetables (in meals), and whole grains/legumes (in meals) and for the TFI we used the chi-squared test (for categorical variables) and Mann-Whitney U test (for numerical variables). Statistical significance was set at p<0.05.

## Results

Of the 1,034 respondents (708 women and 326 men; 68.5 % and 31.5 %, respectively), 593 (57.7 %) had secondary school education, 407 (39.6 %) university education, and only 27 (2.6 %) had primary school education. Most were urban residents (774; 76.3 %). Age distribution (median 38 years; interquartile range 20 years) was relatively uniform, with 14 % younger than 24 years and 36 % older than 45. The median age of women was 38 years (interquartile range 20) and of men 39 years (interquartile range 21).

Table 4 shows the distribution of respondents considering the number of servings/pieces of fruit, meals of vegetables, or meals of cereals/legumes per week. 1025 returned valid FFQ for fruit, 1026 for vegetables, and 1028 for whole grains and legumes (Table 4). Our respondents consumed 6,672 meals of vegetables, 8,167 servings/pieces of fruit, and 2,887 meals of whole grains and legumes a week. Per person it breaks down to 1.18 pieces (about 1 serving) of fruit, 0.93 meals (about 2 servings) of vegetables and legumes, and 0.40 meals (about 1 serving) of cereals and legumes a day. Only 0.2 % of respondents consumed the recommended 3–5 servings of whole grain cereals and legumes per person per week (Table 4). According to our estimations, adult Croatian (Dalmatian) individual consumes about 120 g of fruit, 185 g of vegetables, and 22 g of whole cereals and/or legumes per day.

**Table 4:** Total weekly consumption of fruit (in pieces or servings), vegetables (in meals), and whole grains/legumes (in meals) reported by all respondents

	0/week	1 to 6/week	7 to 10/week	11 to 15/week	16 to 20/week	21 to 25/week	>25 /week	p*
**Fruit**								
All respondents (N=1025)	21 (2.0 %)	488 (47.6 %)	337(32.9%)	90 (8.8 %)	51 (5.0%)	14(1.4%)	24 (2.3 %)	
Women (N=701)	7(1.0%)	322 (45.9 %)	245 (35.0 %)	65 (9.3 %)	36(5.1 %)	10(1.4%)	16 (2.3 %)	**0.009**
Men (N=324)	14 (4.4 %)	166 (51.9%)	92 (28.8 %)	25 (7.8 %)	15(4.7%)	7 (2.2 %)	1 (0.3 %)	
**Vegetables**								
All respondents (N=1026)	6 (0.6 %)	509 (49.3 %)	420 (40.7 %)	68 (6.6 %)	15(1.5%)	13(1.3%)	2 (0.2 %)	
Women (N=702)	5(1.5%)	165 (50.9 %)	123 (38.0 %)	22 (6.8 %)	4(1.2 %)	4(1.2%)	1 (0.3 %)	0.116
Men (N=324)	1 (0.1 %)	344 (49.3 %)	297 (42.6%)	46 (6.6 %)	4 (0.6 %)	5 (0.7 %)	1 (0.1 %)	
**Whole grain/legumes**								
All respondents (N=1028)	275 (26.8 %)	601 (58.5 %)	141 (13.7%)	6 (0.6 %)	3 (0.3 %)	1 (0.1 %)	1 (0.1 %)	
Women (N=704)	106 (32.3 %)	177(54.0%)	36(11.0%)	8 (2.4 %)	1 (0.3 %)	0 (0.0 %)	0 (0.0 %)	**0.028**
Men (N=324)	169 (24.0 %)	424 (60.2 %)	105 (14.9 %)	2 (0.3 %)	2 (0.3 %)	1 (0.1 %)	1 (0.1 %)	

*Chi-square; significant gender differences (p<0.05) are in boldface

Table 5 shows the results of dietary fibre intake based on food consumption data from the first stage of the study, the data obtained from shopping centres about top-selling items in our food groups, and conversion factors calculated as described in the Methods section. The TFI for mixed Croatian adult population was 11.41 g (11.77 g and 10.58 g for women and men, respectively). According to surveys from different European countries (3, 29, 30), TFI from these main fibre sources accounts for 80 % of daily TFI from all sources (which includes processed food and other sources).

**Table 5:** Reported consumption and calculated TFI based on reported intake and conversion factors for fruit, vegetables, and whole grains/legumes

	Fibre-rich food intake (portions/servings/meals)					Dietary fibre intake (g)			
	Total (sum)		Per person		Total (sum)		Per person		p*
	Week	Daily	Week	Daily	Week	Daily	Week	Daily	
**Fruit (pieces)**									
All respondents (N=1025)	8167.00	1166.72	7.96	1.14	24909.35	3558.48	24.28	3.47	
Women (N=702) Men (N=324)	5785.00 2382.00	826.43 340.29	8.24 7.35	1.18 1.05	17644.25 7265.10	2520.61 1037.87	25.13 22.42	3.59 3.20	**0.003**
**Vegetables (meals)**									
All respondents (N=1026)	6672.00	953.14	6.50	0.93	27755.52	3,965.07	27.05	3.86	
Women (N=702) Men (N=324)	4561.00 2111.00	651.57 301.57	6.44 6.52	0.92 0.93	18973.76 8781.76	2,710.54 1,254.54	27.03 27.10	3.86 3.87	0.506
**Whole grains/legumes (meals)**									
All respondents(N=1028)	2887.00	412.43	2.81	0.40	29274.18	4, 182.03	28.48	4.07	
Women (N=704)	2101.00	300.14	2.98	0.43	21304.14	3, 043.45	30.26	4.32	
Men (N=324)	786.00	112.29	2.43	0.35	7970.04	1, 138.58	24.60	3.51	**<0.001**

*Man-Whitney U test; TFI – total fibre intake. Significant gender differences (p<0.05) are in boldface

Women consumed significantly more fruit and whole grains/legumes than men (1.18 vs 1.05 servings/pieces of fruit and 0.43 vs 0.35 meals of whole grains and legumes, respectively) per day. As for vegetables, men consumed slightly more than women (0.93 vs 0.92 meals per day).

## Discussion

Our findings have confirmed our suspicion that Croatian Dalmatian population consumes fibre and fibre-rich foods far below recommended levels, just like many other Western nations (3, 31). An earlier research in Croatia (32) reported average daily intake of dietary fibre of 21 g (recommendations range between 21 and 38 g a day), and 45 % of the intake referred to cereals. However, the relevance of these earlier findings is limited, as the study included a very small sample. Our current findings show that most respondents do not have a sufficient whole grain cereal and therefore fibre intake. Similarly low (about 30 % of the recommended) intakes have been reported by the US Institute of Medicine (14) and the Dietary Guidelines for Americans (7). These two sources also reported that men consumed more cereals than women due to larger bodies and greater need, which contradicts our findings.

What we find discouraging is that 26.8 % of our respondents reported never consuming whole cereals and legumes. This may reflect poor consumer awareness of the health benefits of whole grains (4, 5, 18, 33, 34).

In contrast, only 2.0 % and 0.6 % of our respondents reported never consuming fruit and vegetables, respectively. This may be related to the fact that more than 90 % of our respondents live in the coastal area with a predominant Mediterranean diet, rich in fruit and vegetables (20). However, Mediterranean diet is also rich in cereals and legumes, yet our findings show a very low intake of these. Even the consumption of vegetables and fruit in our adult sample is below the recommended amounts of 200 g for vegetables and 200 g for fruit (13).

Beside the expected difference in the consumption of fruit and whole grain cereals between women and men, our study unexpectedly showed no such gender difference for vegetables, which is in contrast with previous studies (35, 36).

In terms of total fibre intake of 11.4 g in our sample, it is less than half the recommended intake but similar to many European countries (28, 29, 30, 32, 37, 38, 39, 40, 41). As expected from earlier research, it is also lower in men than women. Considering that fruit, vegetables, whole grain cereals, and legumes make 80 % of the TFI (the remaining 20 % is from processed food and other sources) (3, 29, 30), the contribution from fruit and vegetables in our study is 50 % and from whole cereals and legumes 30 %. This latter contribution of only 30 % from whole grains and legumes is quite discouraging, as it is even lower than in other European countries (with more than 40 % contribution) (37, 38, 41, 42).

Our findings, however, should be taken with some reserve due to the following limitations: our participants took the FFQ only once and may have under- or over-reported food intake. Fibre intake may also have been underestimated in calculations, as current conversion factors and nutrition databases may not include all fibre sources and analytical results for whole grain, fruit, and vegetables consumed in Croatia.

## Conclusion

This study gives us a broad idea of the poor dietary habits of Croatian, more specifically Dalmatian population when it comes to fibre-rich foods. It also points to similar deficits in diet of many Western countries and to issues that are deeply rooted in their economies. Raising awareness may sort part of the problem, but unless these deep economic issues are resolved, including high prices of fibre-rich food compared to processed and fibre-poor food and meat, we are afraid health risks related to poor dietary habits will continue. Further research in our country should strive to gain a more accurate and detailed insight into TFI through longitudinal studies of broader samples, using more precise tools.
